# Unusual presentation of polyethylene tibial fracture in a polio patient: a case report

**DOI:** 10.1093/jscr/rjaf084

**Published:** 2025-02-25

**Authors:** Khalid A Alsheikh, Abdullah O Almahayni, Sumaia M Alqaseer, Wafa S Alotaibi, Ziad A Aljaafri

**Affiliations:** Department of Orthopedic Surgery, Ministry of the National Guard – Health Affairs, Riyadh, Saudi Arabia; College of Medicine, King Saud bin Abdulaziz University for Health Sciences, Riyadh, Saudi Arabia; Department of Orthopedic Surgery, Ministry of the National Guard – Health Affairs, Riyadh, Saudi Arabia; Department of Orthopedic Surgery, Ministry of the National Guard – Health Affairs, Riyadh, Saudi Arabia; College of Medicine, King Saud University, Riyadh, Saudi Arabia; Department of Orthopedic Surgery, Ministry of the National Guard – Health Affairs, Riyadh, Saudi Arabia; College of Medicine, King Saud bin Abdulaziz University for Health Sciences, Riyadh, Saudi Arabia

**Keywords:** total knee arthroplasty, polyethylene fracture, complications, polio, case report

## Abstract

The concept of posterior cruciate ligament (PCL)-substituting total knee prostheses has been widely used for total knee arthroplasty (TKA). This design provides better femoral rollback with knee flexion, as it permits more stability under flexion by preventing tibial posterior subluxation. Here, we report a case of an unusual presentation of polyethylene tibial post fracture in a polio patient. Our patient had a history of staged bilateral TKA five years prior to the new presentation. Once the diagnosis was confirmed, the patient was admitted electively for TKA revision and polyethylene exchange. The procedure went well and without complications, and the patient was satisfied upon follow-ups in the clinic.

## Introduction

The concept of posterior cruciate ligament (PCL)-substituting total knee prostheses has been widely used in total knee arthroplasty (TKA) since the original posterior-stabilized condylar prosthesis was introduced in the 1970s [1]. Insall and Burstein [2] incorporated a polyethylene tibial post and a metal femoral cam to serve as a substitute for the PCL. This design provides better femoral rollback with knee flexion as it permits more stability by preventing tibial posterior subluxation [2, 3]. However, despite the advantages of this design, tibial post-wear and deformation have frequently been reported in the literature [4, 5], and previous studies have presented other complications such as breakage (nontraumatic) of the tibial post, tibial post impingement, and patellar clunk syndrome [6, 7].

TKA in polio patients is considered a particularly challenging procedure, but it has been shown to be successful in improving the function of the knee and the quality of life of patients following surgery [8]. Here, we report a case of an unusual presentation of polyethylene tibial post-fracture in a polio patient.

## Case report

**Figure 1:**
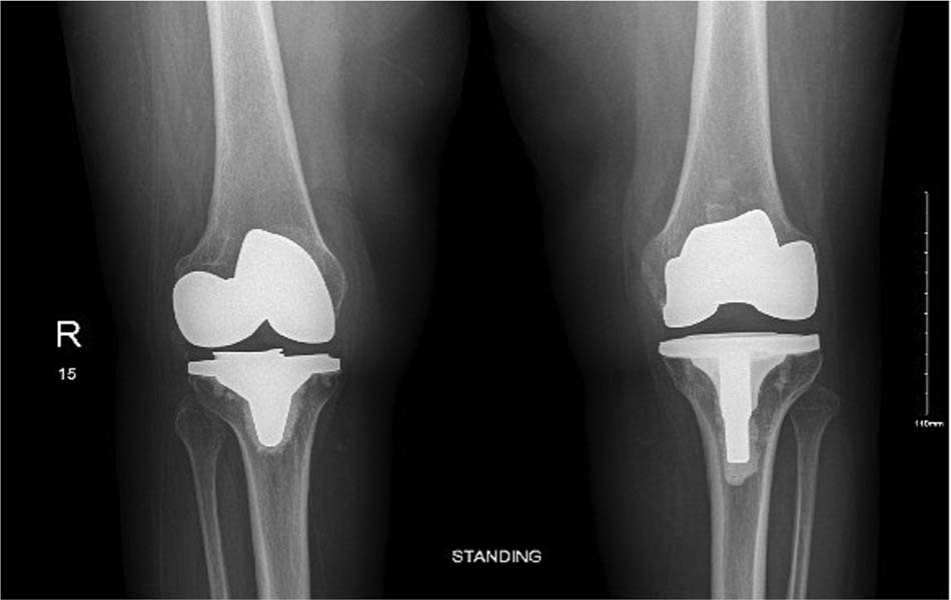
Anteroposterior view of both knees post bilateral staged total knee arthroplasty.

The case involves a 48-year-old obese woman with a body mass index (BMI) of 42, a known medical history of polio, and a baseline of bilateral knee hyperextension of 5º, who underwent bilateral staged TKA with posterior stabilized knee prosthesis in 2018 with a good functional level and outcome (Fig. 1).

The patient presented to the emergency department in October 2023, complaining of severe left knee pain and limited range of motion (ROM) that had been affecting the medial aspect of the knee for 2 days. The pain was sudden, with no history of trauma or previous similar complaints. Her previous functional status was independently ambulatory with the use of a cane, but she started using a walker frame after the onset of pain. In her previous outpatient visits, she had complained of chronic mild pain and hyperextension in the left knee. She was followed in the clinic and used a hinged knee immobilizer for some time. She had gained a significant amount of weight over the previous 5 years, as her BMI was 31 at the time of the TKA but had increased to 42 when she presented in 2023.

**Figure 2:**
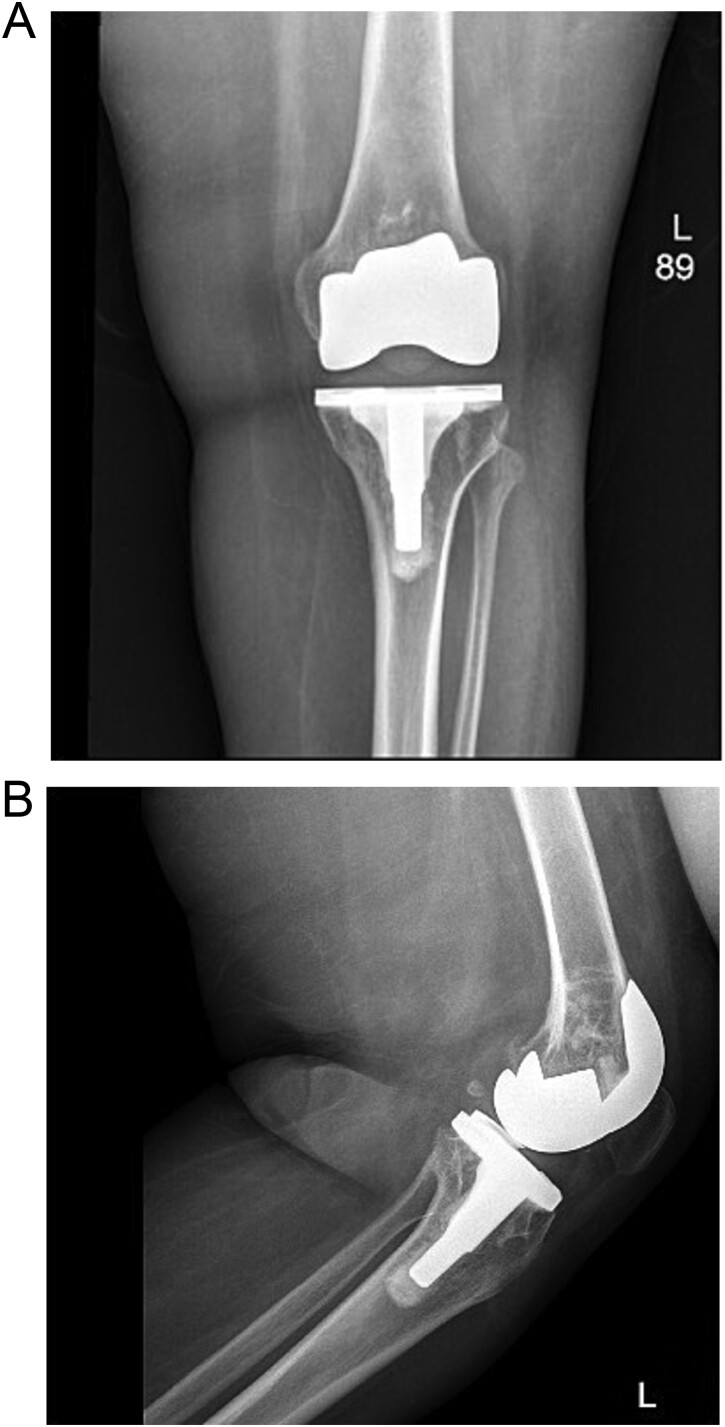
(A) Anteroposterior view of the left knee in the emergency department. (B) Lateral view of the left knee in the emergency department.

Upon examination in the emergency room, the patient was vitally stable, alert, and oriented. Her skin was intact, showing a healed wound. Mild swelling was noted over the left knee joint, with no erythema or ecchymosis. She had tenderness over the medial surface of the knee. The active ROM was from 0º to 90º and was limited due to pain; the passive ROM was from 0º to 110º with a clicking sound during flexion. Soft compartments were noted, and distal neurovascular examination was unremarkable (Fig. 2).

**Figure 3:**
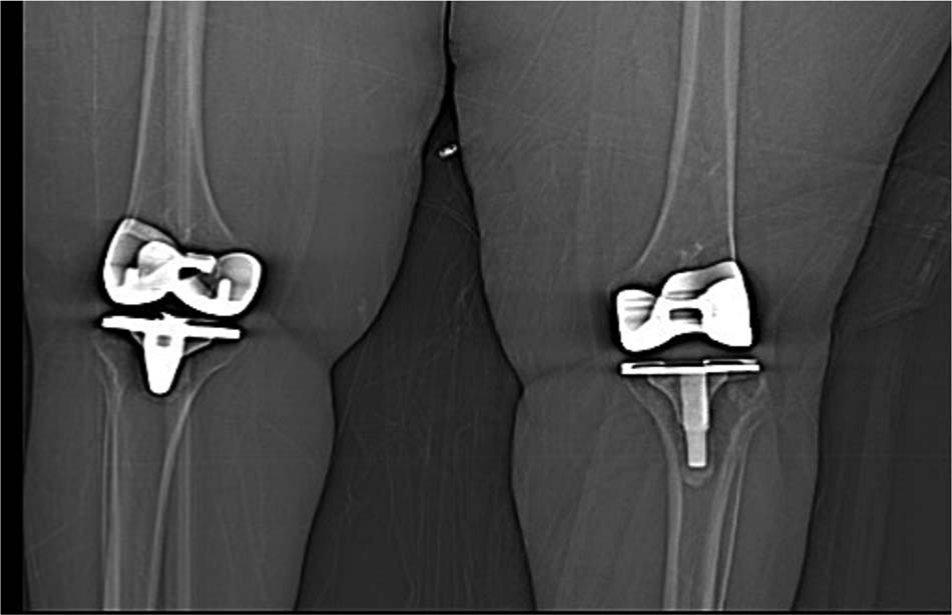
Computed tomography (CT scan) of bilateral knees showing no abnormality.

**Figure 4:**
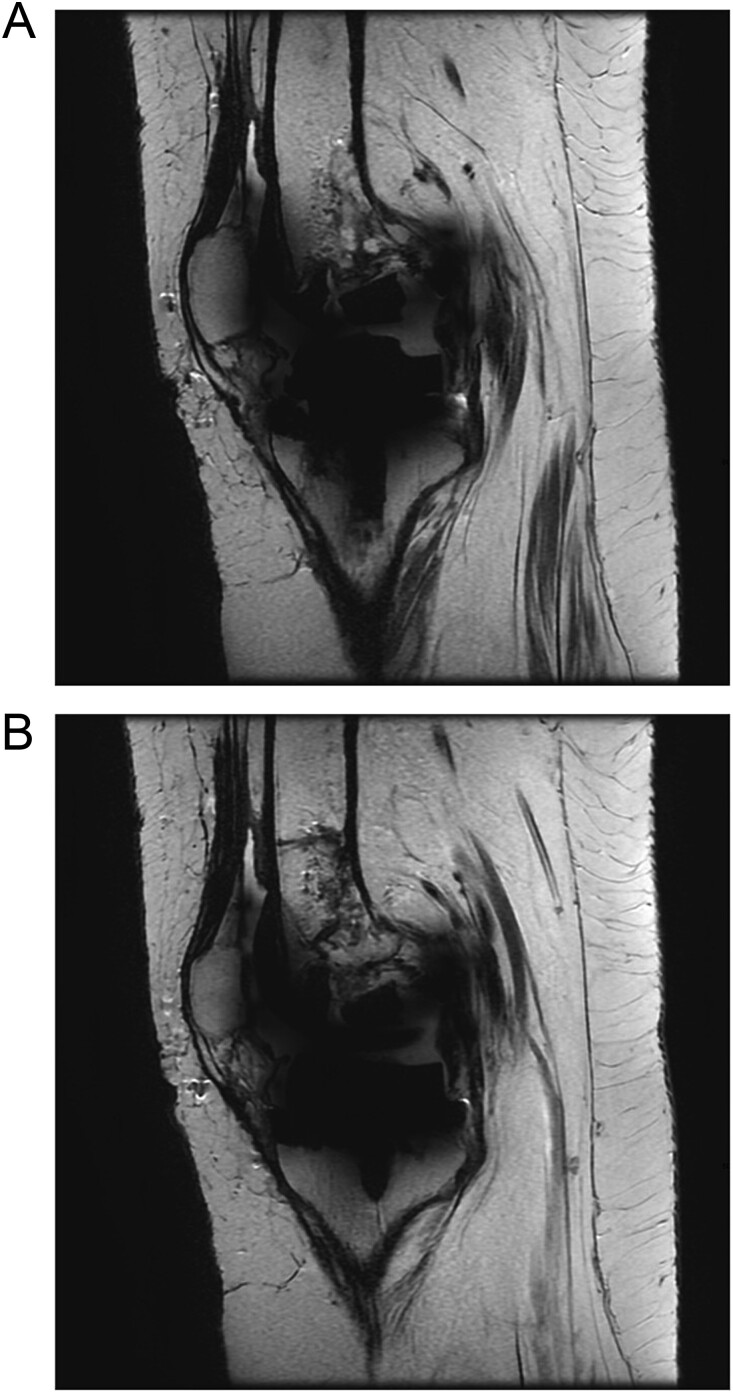
(A and B) Sagittal cuts from the MRI.

The patient was admitted for investigation. Routine radiographic imaging was unremarkable. Magnetic resonance imaging (MRI) and computed tomography scans were conducted and revealed no abnormalities (Figs 3 and 4). Afterward, the patient was examined under anesthesia, where a stable joint was noted with full knee ROM. The only remarkable finding was the clicking sound with flexion. The impression was that there was polyethylene wear due to the significant weight gain, and the decision was made to discharge the patient with instructions to lose weight and to re-admit electively for TKA revision and possible polyethylene exchange if there was no improvement.

**Figure 5:**
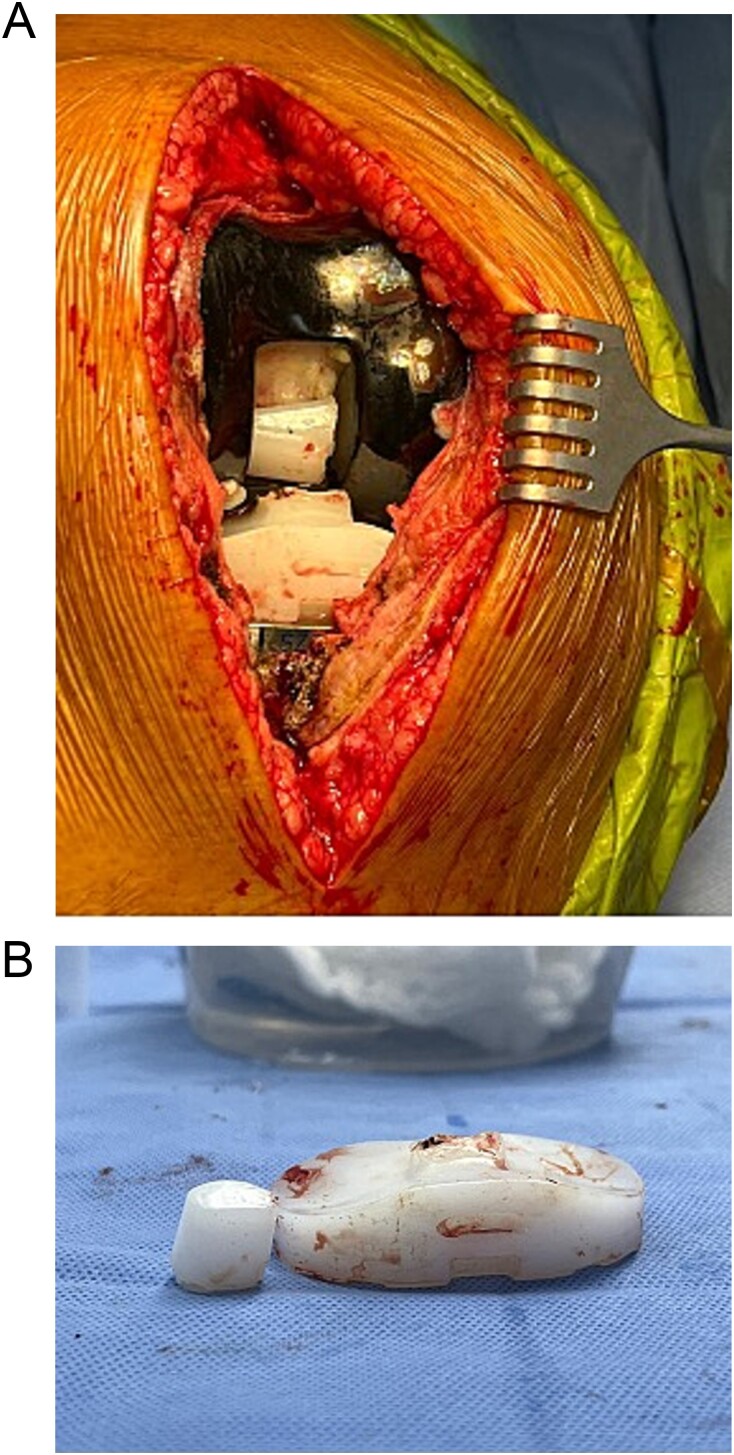
(A) Intraoperative findings showing polyethylene fracture. (B) Fractured polyethylene piece.

The patient was re-admitted for revision TKA involving possible polyethylene exchange. Intra-operatively, the polyethylene insert was found in its place with the tibial post-fractured (Fig. 5). The femur and tibial components were stable, with no signs of wear or loosening. The fractured polyethylene was exchanged with a new and larger one (the new size was 15 compared to the previously used size 11). The knee was examined, and stability was observed throughout the full ROM, with baseline slight hyperextension.

**Figure 6:**
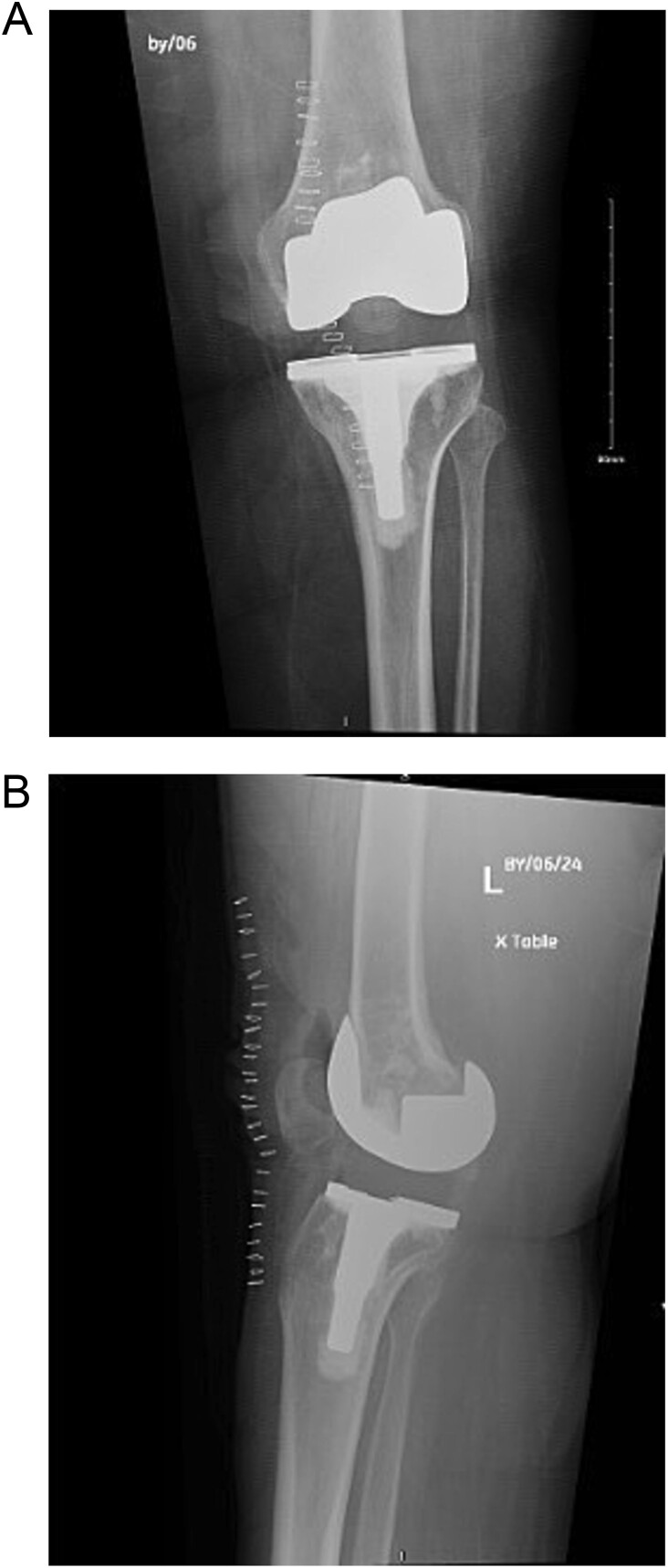
(A) Anteroposterior view of the left knee post revision and polyethylene replacement. (B) Lateral view of the left knee post revision and polyethylene replacement.

Post-operatively, the patient was seen by a physiotherapist and started immediate weight-bearing and range-of-motion exercises. She was seen in the clinic after 6 weeks, and reported significant relief of symptoms and satisfaction with the outcome (Fig. 6).

## Discussion

Polyethylene post-fracture is a rare complication of posterior stabilized TKA that requires surgical intervention with either PS liner exchange to a larger size or upgrading the design to a constrained condylar or rotating hinged component [1]. Diagnosis of polyethylene fracture is a crucial step in its management, but it can be challenging due to its vague presentation and similarity to other more common complications of PS TKA, such as patella clunk syndrome. Most patients with polyethylene fracture report feelings of instability, whereas in this case, our patient did not complain of instability, and her examination under anesthesia showed a stable knee throughout its ROM [1].

Although polyethylene fracture is diagnosed clinically, some imaging modalities are being used to aid in diagnosis. One case reported that taking a lateral radiograph of the knee while in extension may show a posterior displacement of the femur, which may confirm the diagnosis of post-fracture [9]. In addition, MRI may be used to diagnose polyethylene fractures. Although several cases in the literature have done this, most cases with positive MRI results have shown fractured polyethylene intraoperatively [10]. In our case, the MRI possibly did not show the post-facture as the post was not displaced, making it difficult to be seen.

In terms of the predisposing factors for polyethylene post fracture, the literature suggests these are secondary to TKA implant malpositioning but include increased femur component flexion, anterior position of tibial post, valgus knee alignment, high tibial slope, knee hyperextension, and alteration of the joint line by more than 8 mm [1, 9].

Patient-specific factors are not well-studied or described in the literature. In this case, our patient was known to have poliomyelitis, and TKA in patients with poliomyelitis is considered complex and challenging due to muscle weakness and possible ligamentous instability. While there is no preferred design in the literature for patients with poliomyelitis, it is reported that more constrained designs show superior stability and have fewer complications [11]. The fact that this patient had poliomyelitis could be one of the predisposing factors to the increased stress on the polyethylene, which led to the fracture. Having a baseline of 5º of knee hyperextension is another predisposing factor related to this case, as knee hyperextension is a contributing factor to polyethylene wear and fracture [1]. Interestingly, a recent report described a polyethylene (PE) fracture in a poliomyelitis patient with a profile similar to ours. The patient experienced a broken liner in the unafflicted limb, likely due to increase compressive and shear stresses which may have resulted from compensating for weakness in the contralateral limb. This highlights the increased risk of PE fractures in patients with poliomyelitis. Lee *et al*. discussed the importance of carefully selecting the PE material, as it may play a crucial role in ensuring stability in such cases [12].

Another factor specific to this patient is her rapid increase in BMI, from 31 when she underwent the index TKA surgery to 42 five years later. It was noticed in the previously reported cases of polyethylene fractures that the majority involved obese patients [1].

## Conclusion

Polyethylene tibial fractures can occur with an unusual presentation. Therefore, a high level of suspicion for such cases should be maintained, especially in patients with a new onset of immobility or pain during movement. Our patient underwent a polyethylene exchange and was satisfied with the outcome.
